# Global expression analysis of nucleotide binding site-leucine rich repeat-encoding and related genes in Arabidopsis

**DOI:** 10.1186/1471-2229-7-56

**Published:** 2007-10-23

**Authors:** Xiaoping Tan, Blake C Meyers, Alexander Kozik, Marilyn AL West, Michele Morgante, Dina A St Clair, Andrew F Bent, Richard W Michelmore

**Affiliations:** 1 The Genome Center, University of California, Davis, California 95616, USA; 2 Department of Plant and Soil Sciences, University of Delaware, Delaware Biotechnology Institute,15 Innovation Way, Newark, Delaware 19711, USA; 3 Department of Plant Sciences, University of California, Davis, California 95616, USA; 4 Dipartimento di Scienze Agrarie ed Ambientali, Universitá degli Studi di Udine, Via delle Scienze 208, I-33100 Udine, Italy; 5 Department of Plant Pathology, University of Wisconsin, Madison, Wisconsin 53706, USA

## Abstract

**Background:**

Nucleotide binding site-leucine rich repeat (NBS-LRR)-encoding genes comprise the largest class of plant disease resistance genes. The 149 NBS-LRR-encoding genes and the 58 related genes that do not encode LRRs represent approximately 0.8% of all ORFs so far annotated in Arabidopsis ecotype Col-0. Despite their prevalence in the genome and functional importance, there was little information regarding expression of these genes.

**Results:**

We analyzed the expression patterns of ~170 NBS-LRR-encoding and related genes in Arabidopsis Col-0 using multiple analytical approaches: expressed sequenced tag (EST) representation, massively parallel signature sequencing (MPSS), microarray analysis, rapid amplification of cDNA ends (RACE) PCR, and gene trap lines. Most of these genes were expressed at low levels with a variety of tissue specificities. Expression was detected by at least one approach for all but 10 of these genes. The expression of some but not the majority of NBS-LRR-encoding and related genes was affected by salicylic acid (SA) treatment; the response to SA varied among different accessions. An analysis of previously published microarray data indicated that ten NBS-LRR-encoding and related genes exhibited increased expression in wild-type Landsberg *erecta *(L*er*) after flagellin treatment. Several of these ten genes also showed altered expression after SA treatment, consistent with the regulation of *R *gene expression during defense responses and overlap between the basal defense response and salicylic acid signaling pathways. Enhancer trap analysis indicated that neither jasmonic acid nor benzothiadiazole (BTH), a salicylic acid analog, induced detectable expression of the five NBS-LRR-encoding genes and one TIR-NBS-encoding gene tested; however, BTH did induce detectable expression of the other TIR-NBS-encoding gene analyzed. Evidence for alternative mRNA polyadenylation sites was observed for many of the tested genes. Evidence for alternative splicing was found for at least 12 genes, 11 of which encode TIR-NBS-LRR proteins. There was no obvious correlation between expression pattern, phylogenetic relationship or genomic location of the NBS-LRR-encoding and related genes studied.

**Conclusion:**

Transcripts of many NBS-LRR-encoding and related genes were defined. Most were present at low levels and exhibited tissue-specific expression patterns. Expression data are consistent with most Arabidopsis NBS-LRR-encoding and related genes functioning in plant defense responses but do not preclude other biological roles.

## Background

Over 40 plant resistance (*R*) genes that are effective against diverse pathogens and pests, including bacteria, fungi, viruses, nematodes, and insects, have been cloned from both monocot and dicot plant species. These *R *genes can be divided into at least five classes based on the structure of their encoded proteins [1-4]. Genes encoding nucleotide binding site-leucine rich repeat (NBS-LRR) proteins are the most prevalent and can be divided into two major groups based on the encoded N-terminal domains and differences in the NBS domain, as well as several subgroups [5-8]. One major group predominantly encodes a coiled coil domain at the N-terminus (CC-NBS-LRR or "CNL"; e.g. *RPS2 *and *RPM1*), whereas the other group has an N-terminal domain with similarity to the cytoplasmic domain of Drosophila and human Toll-like receptor (Toll-interleukin-1 receptor-like (TIR) domain; TIR-NBS-LRR or "TNL"; e.g. *L6*, *N *and *RPP5*). In the Arabidopsis Col-0 genome 149 NBS-LRR-encoding genes (55 CC-NBS-LRR and 94 TIR-NBS-LRR) and an additional 58 related genes that do not encode LRRs have been identified [7,9]. Based on phylogenetic analysis, protein motif comparisons, and intron positions, four CNL subgroups, eight TNL subgroups, and a pair of divergent "NL" proteins have been identified in Arabidopsis [7,10]. These NBS-LRR-encoding genes are distributed as single genes, clusters, and superclusters in plant genomes [5,7,10,11].

Disease resistance is the predominant function so far demonstrated for plant NBS-LRR-encoding genes [2]. How NBS-LRR proteins function in disease resistance is still under investigation [8]. In addition to directly detecting pathogen ligands, R proteins may also monitor ('guard') the status of the targets of pathogen virulence effectors, or the cellular consequences of the actions of these proteins [2,12-16]. The LRR domains may be involved in protein-protein interactions and at least partly determine resistance specificity [17-27]. Polymorphism in the TIR region has also been shown to affect resistance specificity [23,28]. In addition to their role in determining the recognition specificity, the LRR domains may also participate in defense signaling through both intra- and inter-molecular interactions [27,29-32]. NBS regions contain several conserved motifs and are homologous to the NB-ARC (nucleotide binding domain shared by *Apaf-1*, some *R *genes and *Ced-4*) domain of some eukaryotic cell death effectors such as *Apaf-1 *and *Ced-4 *[33]. The NBS domains of two NBS-LRR proteins, tomato I2 and Mi-1, have been demonstrated to be able to bind and hydrolyze ATP [34] and the ATP binding form is the active configuration of the I2 protein [35], suggesting that the NBS domain functions as a molecular switch in signal transduction eliciting the defense response. The role of CC-NBS and TIR-NBS proteins that lack an LRR domain is unknown but they may function as adaptor proteins similar to Myd88 in mammalian systems [9].

Over 14 NBS-LRR-encoding genes that confer resistance against bacterial, Oomycete, fungal, or viral pathogens have been isolated from Arabidopsis (Table 1). The majority of the subgroups of NBS-LRR-encoding genes contain at least one known *R *gene or its closest homolog in the Col-0 genome, suggesting that the majority of NBS-LRR-encoding genes could be involved in resistance. However, some of the smaller and more divergent subgroups do not contain a known resistance gene and there is limited evidence for the involvement of NBS-LRR proteins in other aspects of plant biology, such as plant development. A T-DNA insertion mutant of an Arabidopsis TIR-NBS-LRR-encoding gene has altered shade avoidance as well as disease susceptibility [36]. The adenylyl cyclase (*AC*) gene cloned from maize pollen plays a role in pollen-polarized growth, for example, and has sequence similarity to NBS-LRR-encoding genes [37]. The tomato *I-2 *gene (CNL type) is expressed at the site of lateral root formation suggesting that it may have functions in addition to pathogen recognition [38]. The other four protein classes that include *R *gene products also contain proteins that participate in other processes, such as two receptor-like kinases, CLAVATA1 and brassinosteroid insensitive1 (BRI1), that are involved in development and hormone reception, respectively, and another receptor-like protein, CLAVATA 2, which functions in plant development [39-42]. Analysis of the expression patterns of NBS-LRR-encoding genes in Arabidopsis may provide clues as to their functions.

**Table 1:** Fourteen NBS-LRR-encoding disease resistance genes cloned from Arabidopsis

**Gene**	**Gene or closest homolog in Col-0**	**Class**	**Pathway**	**Pathogen**	**Avr gene**	**Reference**
*RPM1*	At3g07040	CNL	NDR1	*Pseudomonas syringae*	*avrRpm1*, *avrB*	[43, 117-120]
*RPS2*	At4g26090	CNL	NDR1	*Pseudomonas syringae*	*avrRpt2*	[119, 121-124]
*RPS4*	At5g45250	TNL	EDS1	*Pseudomonas syringae*	*avrRps4*	[100, 125, 126]
*RPS5*	At1g12220	CNL	NDR1	*Pseudomonas syringae*	*avrPphB*	[29, 119, 127, 128]
*RPP1*	At3g44670	TNL	EDS1	*Hyaloperonospora parasitica*	*ATR1*^*NdWsB*^	[19, 120, 129]
*RPP4*	At4g16860	TNL	EDS1(partial NDR1 in cotyledon)	*Hyaloperonospora parasitica*		[119, 126, 130]
*RPP5*	At4g16950	TNL	EDS1	*Hyaloperonospora parasitica*		[46, 126]
*RPP8*/*HRT*/*RCY1*	At5g43470	CNL	Non-EDS1, Non-NDR1	*Hyaloperonospora parasitica*; turnip crinkle virus; cucumber mosaic virus		[20, 126, 131-133]
*RPP13*	At3g46530	CNL	Non-EDS1, Non-NDR1	*Hyaloperonospora parasitica*	*ATR13*	[134-136]
*RRS1-R*	At5g45260	TNLW	Partially NDR1	*Ralstonia solanacearum*	*popP2*	[137, 138]
*RPP2A *and *RPP2B*	At4g19500 At4g19510	TNTNL TNL		*Hyaloperonospora parasitica*		[139]
*RAC1*	At1g31540	TNL	EDS1	*Albugo candida*		[53]
*ADR1*	At1g33560	CNL		*Hyaloperonospora parasitica *and *Erysiphe cichoracearum*		[140]
*RLM1*	At1g64070 and At1g63880	TNL		*Leptosphaeria maculans*		[141]

Although an increasing number of *R *genes are being cloned, little is known about the regulation of plant *R *gene expression. RNA gel-blot analyses have detected low levels of transcripts for most cloned *R *genes in unchallenged plants [1,38,43-48]. However, the expression of few *R *genes has been investigated in detail. Seven TIR-NBS-LRR-encoding *R *genes (*L6, Rpp5, N, M, RPS4, RAC1*, and *Bs4*) have been shown to encode two or more transcripts [46,49-54]; however, the role of alternative splicing in disease resistance is unknown. The alternative transcripts of tobacco *N *gene and Arabidopsis *RPS4 *are known to be important for the defense responses mediated by these two *R *genes [50,52]. Rare alternative splicing has been found for CC-NBS-LRR-encoding *R *genes. In common bean, the alternative transcripts were identified for CC-NBS-LRR-encoding gene *JA1tr *and the alternative splicing is not regulated by pathogen infection [55]. Induction of resistance gene expression by pathogen infection has only been reported for a very small number of *R *genes, such as sugar beet *Hs1*^*pro*-1^, barley *Mla*, rice *Xa1*, and *Xa27 *[56-59]. The induction of a recently cloned rice *Xa27 *gene, encoding a protein with no homology with other R proteins, at the site of pathogen infection is correlated with resistance [59]. The expression of some NBS-LRR-encoding *R *genes have been shown be affected by factors other than pathogen infection, such as tissue type, developmental stage, or environmental conditions [38,60-62].

The methods for analysis of gene expression have advanced from single-gene approaches to a variety of global transcript profiling technologies [63,64]. Large scale expressed sequence tag (EST) sequencing [65] and serial analysis of gene expression (SAGE, [66]) allow quantitative evaluation of gene expression but are less informative than massively parallel signature sequencing (MPSS; [67,68]). MPSS generates millions of tags proximal to the 3' ends of transcripts in a stoichiometric manner; therefore the relative abundance of each transcript can be assessed in each sample and rare transcripts and previously unidentified genes can be detected. It is, however, costly and few samples can be analyzed. Microarrays allow an intermediate number of samples to be analyzed but require *a priori *knowledge of the genes expressed unless tiling arrays are used [69,70]. Current challenges in using microarray analysis include application of appropriate statistical approaches to identify significant changes in expression and making informative comparisons across diverse microarray datasets as well as integrating the microarray data with expression information derived from other approaches [71,72].

In this paper, we describe multiple genomic approaches to characterize the expression of NBS-LRR-encoding and related genes in Arabidopsis. These approaches included representation in EST libraries, MPSS, microarray experiments, gene trap lines and RACE-PCR. The transcript structure was defined for over 80 genes. We determined the level, tissue specificity and possible inducibility of expression for ~170 NBS-LRR-encoding and related genes. Most of the NBS-LRR-encoding and related genes investigated were expressed at low levels and with variable tissue specificities. As previously observed for known *R *genes, expression of these genes was induced during the plant defense response in only a minority of the cases examined. This study provides the foundation for further functional analysis of individual genes.

## Results

### Representation in Expressed Sequence Tag (EST) libraries

We examined the frequency with which NBS-LRR-encoding transcripts were present in EST collections at several times during our study. In April 2002, a total of 181,406 Arabidopsis sequences from the NCBI EST database were searched for similarity to the spliced genomic ORFs and genomic sequences of 170 NBS-LRR-encoding and related genes. ESTs were detected for about half (98) of these 170 genes; most genes (81) had five or less representatives per gene. At these low frequencies other expressed NBS-LRR-encoding and related genes could have gone undetected in this depth of EST sampling. Therefore more efficient and sensitive methods were used to analyze the expression of NBS-LRR-encoding and related genes. When the same analysis was repeated in July 2006, 622,792 Arabidopsis EST sequences were searched for similarity to 172 NBS-LRR-encoding and related genes. ESTs were detected for only about two thirds (120) of the 172 genes analyzed. Most of these (94) still had ten or fewer representatives per gene (Table 2; detailed information in additional file 1 and online database [73]).

**Table 2:** Summary of EST, RACE, MPSS, and microarray expression analyses of NBS-LRR-encoding and related genes

**Predicted Protein Domains **^**a**^	**Code**	**# based on prior annotation**	**# based on full manual annotation**	**ESTs (# expressed/# studied) 2002**	**ESTs (# expressed/#studied) 2006**	**RACE (# expressed/# studied)**^**b**^	**MPSS (# expressed/# studied)**	**Microarray (#P or M/# studied)**^**c**^	**# detected by both MPSS and microarray**
CC-NBS-LRR	CNL	48	51	28/49	36/51	26/35	46/49	32/47	31
NBS_CC_-LRR	NL	2	4	2/4	3/4	1/1	3/4	2/3	2
TIR-NBS-LRR	TNL	82	83	46/83	56/83	38/50	79/83	50/80	50
NBS_TIR_-LRR	NL	2	2	0/2	0/2	0/1	1/2	0/2	0
TIR-NBS-LRR-X	TNLX	5	5	3/5	4/5	4/5	5/5	3/5	3
TIR-NBS-TIR-NBS-LRR	TNTNL	2	2	2/2	2/2	2/2	2/2	1/2	1
TIR-TIR-NBS-LRR	TTNL	0	2	2/2	2/2	2/2	2/2	2/2	2
*Total with LRRs*		*141*	*149*						
TIR-NBS	TN	14	21	9/15	10/15	5/9	13/15	10/15	10
TIR-X	TX	23	30						
X-TIR-NBS-X	XTNX	0	2	2/2	2/2		2/2	1/2	1
CC-NBS	CN	4	4	3/4	3/4	2/2	4/4	1/2	1
CC-NBS-X	CNX	1	1	1/1	1/1	1/1	1/1	1/1	1
CC (related to CNL)	C	0	1						
NBS_CC_	N	1	1	0/1	1/1	0/1	1/1	1/1	1
*Total without LRRs*		*43*	*58*						
Total			207	98/170	120/172	81/109	159/170	104/162	103

^a ^"CC" and "TIR" subscript indicates motifs predictive of a CC or TIR domain N-terminal to the NBS. The first four columns are from [7].

^b ^NBS-LRR-encoding and related genes for which RACE-PCR products were detected for either 5' or 3'end, or both.

^c ^Number called as present (P) or marginal (M) in one of the three control Col-0 samples collected 4 hours post treatment with 0.02% silwet (experiment described in [79]; additional file 1). ATH1 array was used for transcript profiling.

### Massively Parallel Signature Sequencing (MPSS) analysis

The expression of 170 NBS-LRR-encoding and related genes was then determined by utilizing the DuPont MPSS database for Arabidopsis and the public Arabidopsis MPSS database ([74-77]). On average, there were approximately one million tags in each of the 22 DuPont libraries (Table 3) and about two and half million tags in each of the seventeen libraries generated by Meyers et al. (Table 4). These tags represent all the transcripts in a given sample and the frequency of each tag is correlated to the expression level of each represented gene.

**Table 3:** MPSS libraries of *Arabidopsis thaliana *from Dupont MPSS databases

**Library**	**Code**	**Ecotype**	**Tissue**	**Description**	**# signatures **
Ale1lm.1	a	Columbia	Leaf	Early and late leaves	391295
Afl2lm.1	b	Columbia	Flower – shoot	Flower and shoot meristems	1997189
Aro1lm	c	Columbia	Root	Roots	1531770
Ase2lm-ea	d	Columbia	Seed	Early stage developing seeds	1726426
Ase2lm-la	e	Columbia	Seed	Late stage developing seeds	287779
Ase1lm	f	Columbia	Seed	Germinating seeds	1127420
Asegllm	g	Columbia	Seed	gl2 mutant seed, 7 DAF	1572064
Asd2lm-t1.1	h	Columbia	Seedling	Top part of seedlings grown without sucrose	1863086
Asd2lm-t2.1	i	Columbia	Seedling	Top part of seedlings grown with sucrose	1793820
Asd2lm-b1.1	j	Columbia	Seedling	Bottom part of seedlings grown without sucrose	870914
Asd2lm-b2.1	k	Columbia	Seedling	Bottom part of seedlings grown with sucrose	897853
Asdl1lrm.1	l	Columbia	Seedling	Stage 1 seedlings	1152083
Ack1lm-ctrGVG.1	m	Landsberg	Seedling	IPT plants untreated	1893424
Ack1lm-tr6.1	n	Landsberg	Seedling	IPT plants treated with DEX, 6hrs	1639214
Ack1lm-tr24.1	o	Landsberg	Seedling	IPT plants treated with DEX, 24 hrs	1783294
Abawt-ctr	p	Landsberg	Seedling	Wildtype plants, uninduced	1477653
Abawt-tr	q	Landsberg	Seedling	Wildtype plants, ABA induced	1266435
Aabi1-ctr	r	Landsberg	Seedling	abi1 plants, uninduced, 3 and 5 hrs	1656984
Aabi1-tr	s	Landsberg	Seedling	abi1 plants, ABA induced, 3 and 5 hrs	1241725
Afl2lm.test	t	Columbia	Flower – shoot	Flower and shoot meristem	
Ase2lm-ea.test	u	Columbia	Seed	Developing seeds, early stage	
Ase7lm-WT	v	Columbia	Seed	Seed wt, 7 DAF	

**Table 4:** MPSS libraries of *Arabidopsis thaliana *from public MPSS databases

**Library**	**Code**^**a**^	**Ecotype**	**Tissue**	**Description**	**# signatures**
CAF	1	Columbia	Callus	Callus – actively growing	1959539
INF	2	Columbia	Inflorescence	Inflorescence – mixed stage, immature buds	1774306
LEF	3	Columbia	Leaf	Leaves – 21 day, untreated	2884598
ROF	4	Columbia	Root	Root – 21 day, untreated	3642632
SIF	5	Columbia	Silique	Silique – 24 to 48 hr post-fertilization	2012859
AP1	6	Columbia	Inflorescence	ap1-10 inflorescence – mixed stage, immature buds	2964724
AP3	7	Columbia	Inflorescence	ap3-6 inflorescence – mixed stage, immature buds	2435965
AGM	8	Columbia	Inflorescence	agamous inflorescence – mixed stage, immature buds	2575670
INS	9	Columbia	Inflorescence	Inflorescence – mixed stage, immature buds	2890894
ROS	10	Columbia	Root	Root – 21 day, untreated	2458436
SAP	11	Columbia	Inflorescence	sup/ap1 inflorescence – mixed stage, immature buds	2310350
S04	12	Columbia	Leaf	Leaves, 4 hr post SA treatment	3006975
S52	13	Columbia	Leaf	Leaves, 52 hr post SA treatment	2964840
LES	14	Columbia	Leaf	Leaves – 21 day, untreated	3109385
GSE	15	Columbia	Seedling	Germinating seedlings	2550655
CAS	16	Columbia	Callus	Callus – actively growing	1919458
SIS	17	Columbia	Silique	Silique – 24 to 48 hr post-fertilization	2349283

^a^Libraries 1 to 5 were made using the classic MPSS protocol; all subsequent libraries were made using signature MPSS [75, 76].

Most of the 170 NBS-LRR-encoding and related genes were detected in at least one library and at low levels (1 to 991 adjusted parts per million (adjPPM) or transcripts per million (TPM) compared to reference genes such as *EF-1α *(769 to 4061 adjPPM) and *ACT-2 *(6–2925 adjPPM) (reference genes used in [9]). The most highly expressed NBS-LRR-encoding or related gene was At3g04210 at a level of 991 TPM in the library made from leaves 52 hours after treatment with salicylic acid (S52). The second most highly expressed gene (683 TPM) was At1g72900 in the library made from callus (CAS). The gene with highest expression in untreated Arabidopsis tissues was At3g04210, which was expressed at 322 TPM in a library made from leaf (LES). Other genes were expressed at much lower levels than these two genes. About half of the genes (73) were expressed at very low levels of less than 32 adjPPM or TPM. Expression of only 11 genes was not detected in any of the 37 MPSS libraries. The genes exhibiting higher levels of expression in the MPSS analysis, for example At4g33300 and At3g50950, were also well represented in the EST dataset. Expression of 17 of the 21 predicted or potential pseudogenes in Col-0 genome [7] was detected in at least one MPSS libraries generated from tissue of Col-0.

The total number of NBS-LRR-encoding and related genes expressed varied widely between MPSS libraries from 101 detected in the Col-0 leaf library (LEF), to 15 detected in the library made from Col-0 late stage developing seeds (Ase2lm-la). On average, each gene was present in only 15 of the 39 libraries (Additional file 1). The four most prevalent genes, At1g61190, At1g61300, At1g59124, and At3g07040, which all encode CC-NBS-LRR proteins, were detected in 37 out of 39 libraries studied.

Most NBS-LRR-encoding and related genes exhibited different levels of expression in different tissues, at different developmental stages or in different genotypes of Arabidopsis (Additional file 1). Forty NBS-LRR-encoding and related genes were expressed at a higher level in callus than in any other tissues examined in the public Arabidopsis MPSS database. Some genes were preferentially expressed in aerial plant parts (e.g. At5g44870, 58 TPM in leaf and 2 TPM in root), while others were root-specific (e.g. At5g45210, 28 TPM in roots, 0 PPM in leaves and other tissues). Some genes were expressed primarily in flowers (e.g. At1g63740, 52 TPM in flower, 17 TPM in silique, and less than 5 TPM in root, leaf and callus). Some were induced in response to a chemical or hormone (e.g. At1g72850, 12 adjPPM vs 3 adjPPM in the abscisic acid (ABA) induced Landsberg *erecta *(L*er*) plants vs uninduced plants, respectively). This range of expression patterns suggests that NBS-LRR-encoding and related genes may have a variety of functions.

Visual inspection revealed no obvious correlation between the encoded protein structures (CC-NBS (CN), CC-NBS-LRR (CNL), TIR-NBS (TN) or TIR-NBS-LRR (TNL)) of the genes studied and their expression patterns. Each group contained genes with different expression levels and tissue specificities. Within the four CNL subgroups and the eight TNL subgroups of NBS-LRR-encoding genes identified by Meyers et al. [7], a wide variety of gene expression patterns were observed (Additional file 1). Consequently, no correlation was detected between gene expression pattern and position on the phylogenetic tree. There was also no obvious correlation between chromosomal location and expression pattern (Additional file 1).

One hundred and twenty NBS-LRR-encoding or related genes (two divergent NLs, 32 CNLs, two CNs, one NL (CC type), one NBS (CC type), 63 TNLs, five TNLX, two TNTNL, two TTNL, one NL (TIR type), two XTNX, and seven TNs) studied were represented by more than one MPSS tag (Additional file 1). Multiple tags can be detected for one gene when alternative splicing results in different stop codons and polyadenylation sites or when the polyadenylation site varies either side of a *Sau*3A site [9]. A total of 72 genes showed possible alternative polyadenylation. Twelve of these genes showed possible alternative splicing because some of the tags detected were located at splice sites or within introns; 11 out of these 12 genes were of TNLs rather than CNLs, consistent with the multiple introns in TNL-encoding genes and a paucity of introns in CNL-encoding genes. Alternative splicing was confirmed by RACE-PCR and subsequent sequencing for four of these genes: At1g63750, At4g16860 (*RPP4 *homolog in Col-0), At4g16950 (*RPP5 *homolog in Col-0), and At5g46270. The failure to detect alternative transcripts for the other eight genes may have been due to tissue specificity or low abundance of the alternatively spliced transcripts. For other genes with multiple tags detected, some were due to shifts in the polyadenylation site and some were due to the tags representing several members of a gene family.

### Microarray analysis

To examine expression under a different range of conditions than those from which the MPSS libraries had been made, the expression of NBS-LRR-encoding and related genes was also analyzed using data from Affymetrix microarrays that were generated as components of two larger studies [78,79]. Both of these experiments utilized the whole genome array (ATH1; Affymetrix), which contained 152 probe sets representing 162 NBS-LRR-encoding and related genes which were located on the phylogenetic tree generated by Meyers et al. [7], including 13 known resistance genes (*RPP8 *is not represented on this array) or their homologs in Col-0.

In one experiment, changes of expression of NBS-LRR-encoding and related genes in response to application of 0.3 mM salicylic acid (SA) were analyzed in seven Arabidopsis accessions (Col-0, Cvi-1, Est, Kin-0, Mt-0, Tsu-1, and Van-0) as part of a study to identify expression level polymorphisms as described in [79] and ELP website [80]. Re-analysis of this data revealed that approximately two-thirds of the NBS-LRR-encoding and related genes were expressed above the detection threshold in the control Col-0 sample (in at least one of the three replicates collected 4 hours post treatment with 0.02% silwet). The expression of nine genes, At1g57630, At1g59124, At1g72900, At1g72910, At3g04210, At3g50950, At4g16950, At4g33300, and At5g45510 in at least one of the three control Col-0 samples was higher than the average of present signal in the corresponding control Col-0 sample. Based on their expression levels in the control samples, most of the 162 NBS-LRR-encoding and related genes (138 probe sets out of 152 probe sets) exhibit significant differences in their expression levels between at least one pair of Arabidopsis accessions, suggesting the natural variation in expression of NBS-LRR-encoding genes between different accessions.

The expression levels of the majority of NBS-LRR-encoding and related genes were not significantly altered by the SA treatment compared to control samples harvested at the same timepoints; however, the expression of 15 genes (three CNLs (At3g14470, At5g04720, and At5g66900), one CN (At5g45490), one CNX (At5g66630), seven TNLs (At1g17600, At3g44630, At4g12010, At4g16860 (*RPP4 *homolog), At5g36930, At5g41740, and At5g46520), two TNs (At1g66090 and At1g72900), and one divergent NL (At5g45510)) was significantly induced in Col-0 plants 4 hours after SA treatment (Additional files 1 and 2). In addition, the expression of four NBS-LRR-encoding and related genes (two TNLs (At3g44400 and At4g36150), one TN (At3g04210), and one CNL (At3g50950)) was down-regulated in Col-0 plants 28 hours after SA treatment. These four genes also showed increases in their expression 4 hours after SA treatment though not to a statistically significant extent.

The expression of different NBS-LRR-encoding and related genes was affected at different time points after SA treatment in the various Arabidopsis accessions (Additional file 2). A total of 33 probe sets exhibited differential expression in response to SA treatment in at least one Arabidopsis accession. Some NBS-LRR-encoding genes exhibited similar responses to SA treatment across different accessions though some do not. For example, the expression of one CNL-encoding gene, At1g12280, was down-regulated both in Est 4 hours after SA treatment and in Van-0 28 hours after SA treatment. One CNL gene, At4g14610, showed elevated expression 4 hours after SA treatment in Kin-0 and Tsu-1. One CNL gene, At5g66900, showed elevated expression in five Arabidopsis accessions, Col-0, Kin-0, Mt-0, Tsu-1, and Van-0, 4 hours after SA treatment but down-regulated expression 28 hours after SA treatment in Van-0 only.

In order to compare the SA responses to changes of expression during the basal defense response, we reanalyzed the data set on the response to flagellin generated by Zipfel et al. [78] using the same procedures as used above. In this analysis, less than half of the NBS-LRR-encoding and related genes (69) were expressed above the detection threshold in at least one of the two control wild-type Landsberg *erecta *(L*er*) samples. The expression of ten NBS-LRR-encoding and related genes (five CNLs (At3g07040 (*RPM1*), At3g50950, At4g26090 (*RPS2*), At4g33300, and At5g04720), three TNLs (At1g56510, At1g56540, and At5g22690), and two TNs (At1g72900 and At1g72940)) was induced by flagellin treatment in wild-type L*er*. Interestingly, out of these ten NBS-LRR-encoding and related genes, one gene, At1g72900, was also induced in Col-0 and Van-0 4 hours after SA treatment; one gene, At5g04720, was also induced in Col-0 and Mt-0 4 hours after SA treatment; two genes, At4g33300 and At1g56510, were also induced in Mt-0 4 hours after SA treatment; and one gene, At3g50950, exhibited down-regulated expression in Col-0 28 hours after SA treatment, suggesting the interaction between plant basal defense response and SA pathway (Additional file 2).

Overall, the expression patterns of the NBS-LRR-encoding and related genes detected in the above microarray experiments were generally consistent among array experiments. Most genes were detected as expressed at low levels and not induced by treatment with defense signals.

**Figure 1:**
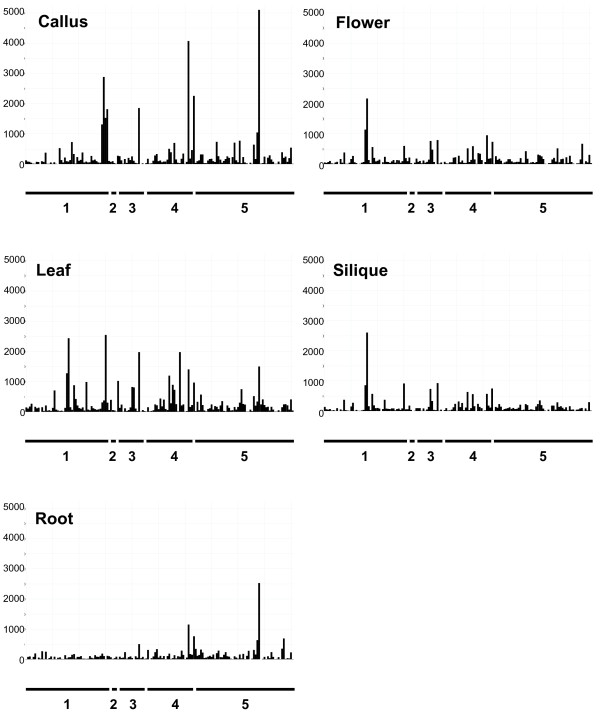
The expression level of 162 NBS-LRR-encoding and related genes in five different tissues of Arabidopsis wild-type Col-0. All NBS-LRR-encoding and related genes are ordered according to their At numbers along the X axis. Each Arabidopsis chromosome is indicated below each graph. Y-axis indicates the relative expression level of each probe set after scaling the mean intensity of each ATH1 microarray to 500.

Five of the mRNA samples that were used to generate the Arabidopsis MPSS libraries described above (LEF, CAF, INF, ROF, and SIF MPSS libraries; [75-77]) were also analyzed using ATH1 arrays to cross-validate the two approaches. The expression patterns of the NBS-LRR-encoding and related genes detected in the microarray experiment were generally consistent with the MPSS data (Table 2; Additional file 1). These 162 genes showed different expression levels in different tissues. For example, both the MPSS data and Affymetrix array data indicated that At4g16990 has its highest expression in leaf, lower expression in flowers and siliques, and its lowest expression in callus and root. Most genes were usually the most highly expressed in callus; expression levels in flowers and siliques were similar (Figure 1). Forty-six of the 67 genes with undetectable levels of expression in the leaf sample in the microarray analysis had 0 MPSS tags in the leaf MPSS library (LEF), while 75 of the 95 genes detected by microarray analysis had more than one tag in the MPSS leaf library. For 88 NBS-LRR-encoding and related genes which are represented by unique MPSS tags and probe sets on the ATH1 array, Spearman rank correlation test showed a good correlation between MPSS data and Affymetrix array data generated from the same leaf tissue (correlation coefficient 0.74357, *P*-value < 0.001).

### Reporter gene traps

We attempted to use enhancer and gene trap lines in parallel to the above global analyses, to gain information on cell-specific expression patterns for individual NBS-LRR-encoding or related genes and to investigate their inducibility in greater detail. Gene traps and enhancer traps contain insertions of the *β*-glucuronidase (*GUS*) reporter gene under the control of no promoter or a minimal promoter respectively [81-83]. The expression pattern of a gene with a reporter gene inserted within it or nearby can be monitored via expression of the reporter gene. Gene trap and enhancer traps also allow the analysis of the mutant phenotypes resulting from the disruption of chromosomal genes [81-84].

**Figure 2:**
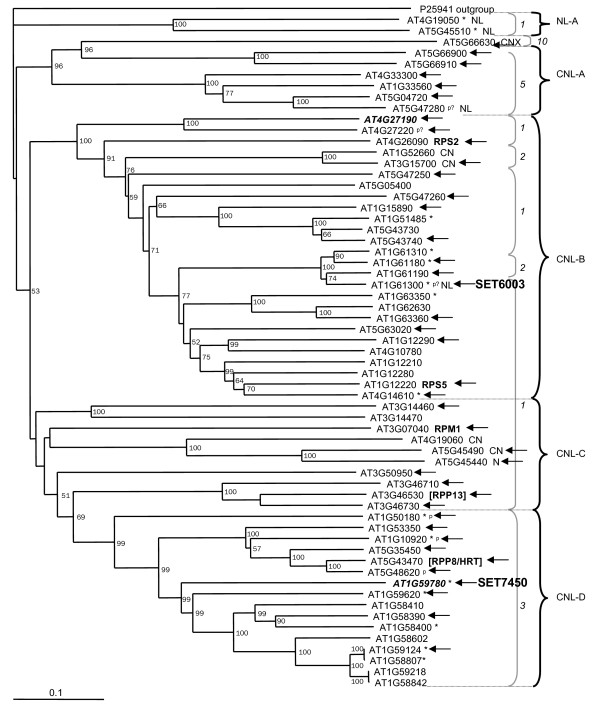
The distribution of CC-NBS-LRR-encoding and related genes analyzed on neighbor-joining tree generated by Meyers et al. [7]. The genes studied by RACE are indicated by black arrows, the genes analyzed using RT-PCR are displayed in bold italic, and NBS-LRR-encoding and related genes with enhancer trap insertions are marked in bold. Other figure denotations are as described in Meyers et al. [7].

**Figure 3:**
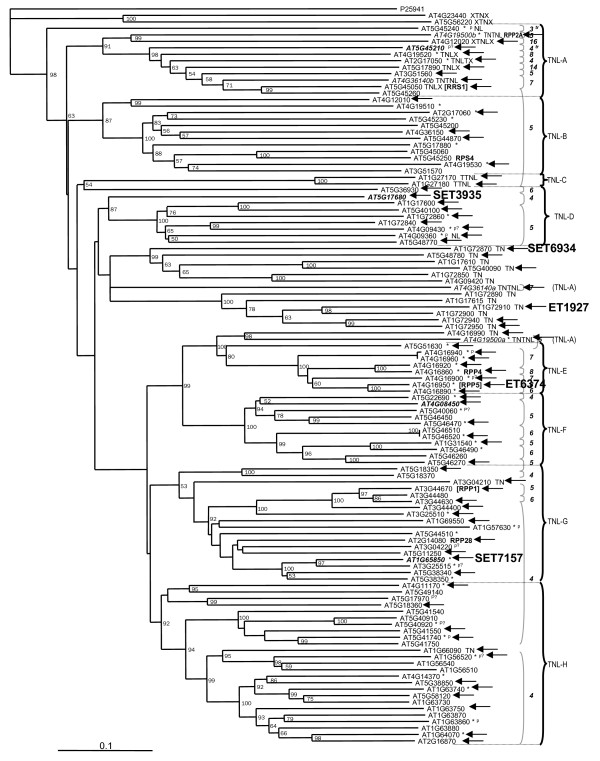
The distribution of TIR-NBS-LRR-encoding and related genes analyzed on neighbor-joining tree generated by Meyers et al. [7]. The genes studied by RACE are indicated by black arrows, the genes analyzed using RT-PCR are displayed in bold italic, and NBS-LRR-encoding and related genes with enhancer trap insertions are marked in bold. Other figure denotations are as described in Meyers et al. [7].

The Cold Spring Harbor database of flanking sequences for Arabidopsis Gene Trap lines [85] and the database of the Ds insertion lines from Singapore IMA (Institute of Molecular Agrobiology, [86]) were searched in 2002 for insertions in NBS-LRR-encoding and related genes using BLAST. Ten enhancer trap lines and three gene trap lines were identified with insertions into NBS-LRR-encoding or related genes. The insertion sites and orientations were confirmed for seven enhancer trap lines with insertions into five NBS-LRR-encoding genes and two TIR-NBS-encoding genes (Table 5, Figures 2 and 3). No gene trap lines were confirmed.

**Table 5:** Summary of enhancer trap lines

**Enhancer trap line**	**Gene interrupted**	**Gene type**	**RACE -PCR**^**a**^	**GUS staining**	**Microarray data**^**b**^	**MPSS data**^**c**^
**SET6934**	At1g72870	TN	ND	no	108.3(M)	1–15(14)
**SET7157**	At1g65850	TNL	ND	no	55.4(A)	3–18(4)
**SET3935**	At5g17680	TNL	ND	no	54.4(A)	3-3(1)
**SET7450**	At1g59780	CNL	ND	no	41.6(A)	3–45(3)
**SET6003**	At1g61300	NL	Expressed	no	552.5(P)(probe set cross-hybridizes to At1g61180, At1g61190, and At1g61310)	4-179(37), MPSS tags also represent At1g61180, At1g61190, and At1g61310.
**ET1927**	At1g72910	TN	Expressed	Weak staining in leaf; weak induction by BTH	5566.9(P) (probe set cross-hybridizes to At1g72930)	2–127(5), two MPSS tags also represent other genes.
**ET6374**	RPP5 (At4g16950 in Col)	TNL	Expressed	no	1377.5(P) (probe set may cross-hybridize to At4g16860, At4g16890, and At4g16920)	3–168(31), several MPSS tags also represent several other genes

^a ^ND = no detectable expression.

^b ^Called present (P), marginal (M), or absent (A) in microarray expression data in control leaf sample of SA experiment [79].

^c ^Range of adjPPM or TPM (# of libraries with expression detected).

**Figure 4:**
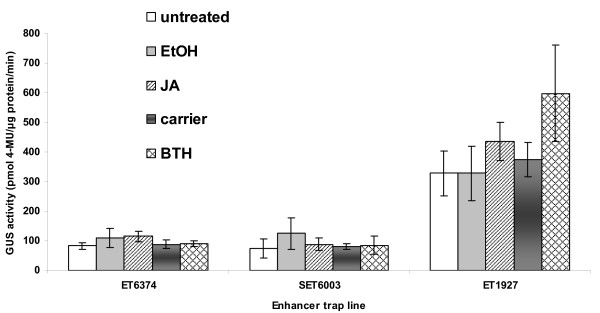
**GUS quantitative assays of enhancer trap lines with insertions into two NBS-LRR-encoding and one related gene**. GUS expression levels of each enhancer trap line untreated or five days after application of JA or BTH. Treatments with ethanol (EtOH) or BTH carrier (carrier) were used as controls. Average GUS expression levels and standard deviations were based on three biological replicates. The background levels of GUS activity in untreated Col-0 plants were below 20 pmol 4-MU/μg protein/min.

The expression pattern of the corresponding NBS-LRR-encoding and related gene in each confirmed enhancer trap line was analyzed using GUS staining and quantitative GUS assays of seedlings and five-week old plants. No GUS activity was detected histochemically in whole seedling, leaves, roots, flowers and stems of any of the five NBS-LRR and one TIR-NBS enhancer trap lines. This indicated that the low levels of expression detected in the microarray and MPSS experiments for these genes were below the detection threshold of GUS assays in these enhancer trap lines. We found no evidence that any specific cell types exhibited localized, high levels of expression as has been observed for insertions into some other types of genes [81,87,88]. In the enhancer trap line with an insertion into a TIR-NBS-encoding gene, At1g72910, there was very faint blue color throughout the leaf after staining for GUS activity. This low level of GUS activity was confirmed using a GUS quantitative assay (Figure 4). At1g72910 was one of the more highly expressed genes detected in the MPSS libraries and microarray data in Col-0 leaves (Table 5). These data indicate the threshold necessary for histochemical detection of gene activity in gene trap lines.

These seven NBS-LRR-encoding and related genes were not induced by either salicylic acid (SA) or flagellin treatments in the above microarray experiments. To investigate the possibility that detectable changes in expression occurred transiently at time points not examined in the microarray experiments, jasmonic acid (JA) or benzothiadiazole (BTH) was applied to each of the enhancer trap plants. Neither GUS staining nor quantitative GUS assays provided evidence of induced expression of any the five NBS-LRR-encoding genes and one TIR-NBS-encoding gene in response to BTH or JA (data not shown). The expression of At1g72910, on the other hand, was induced approximately two-fold five days after treatment with BTH (Figure 4), although significant induction by SA had not been detected in the microarray analysis. To examine the possibility that changes in expression occurred in a restricted number of cells at sites of pathogen penetration, each of the seven enhancer trap plant was challenged by *P. syringae *pv. *tomato *strain DC3000 carrying *avrRpt2 *or *avrRpm1*. All lines showed a similar hypersensitive response (HR) as wild-type Landsberg *erecta *(L*er*) plants, which is the genetic background of the enhancer and gene trap lines. There was no observable localized induction of *GUS *gene expression in the infiltrated area that reacted to the pathogen.

### Rapid Amplification of cDNA Ends (RACE) analysis

The expression of NBS-LRR-encoding and related genes was further studied using RACE-PCR as a sensitive qualitative method to detect expression as well as to define the 5' and 3' ends of the transcripts. Confirmation of transcript boundaries was a prerequisite to studies of gene function because approximately a third of the NBS-LRR-encoding and related genes in the public database were previously annotated incorrectly and therefore had to be corrected by manual re-annotation [7]. The sequences of our 5' and 3' RACE products were compared with the Arabidopsis genomic sequence to determine the initiation and termination sites of each transcript (Additional file 1 and online database [73]).

A total of 109 NBS-LRR-encoding and related genes were analyzed using total RNA extracted from leaves of Col-0 as template. At least one RACE product was detected for 81 genes (for 26 out of 35 CNL genes and for 38 out of 50 TNL genes analyzed; Table 2). Both 5' and 3' products were detected for 68 of these 81 genes. Only 5' products were detected for another six genes and only 3' products for an additional seven genes. Neither 5' nor 3' products were detected for the other 28 genes; the lack of expression of six of these 28 genes in leaves was confirmed using RT-PCR with primers that should have amplified an internal region of the transcript (Figures 2 and 3, Table 2). RACE for six NBS-LRR-encoding genes that were not detectable in leaves from four-week old plants was also conducted using total RNA extracted from seven-day old seedlings as templates. RACE again failed to detect expression of five of these genes, At2g17050, At3g46730, At4g08450, At4g09360, and At4g27190. RACE products were only detected for At1g65850 in seedlings. For two NBS-LRR-encoding genes, At3g44400 and At4g33300, RACE was also performed using the total RNA extracted from flowers as templates in addition to the templates generated from leaf and young seedlings. The RACE results revealed that the expression of these two NBS-LRR-encoding genes showed different expression pattern in leaves, seedlings, and flowers. At4g33300 was expressed highest in seedlings, and then the flower and leaf tissues, while At3g44400 was expressed higher in leaves than in flowers.

For the above RACE-PCR products, the length of the 5' UTR ranged from zero to 241 bp (average = ~50 bp); most 5' transcription start sites were within 100 bp of the ATG start codon. There was some variation in the transcription initiation site and often multiple initiation sites were detected for each gene. The length of the 3' UTR ranged from 6 bp to 896 bp and most 3' UTRs were several hundred bp long (average = ~250 bp). The most common variation in 3'UTRs was due to alternative polyadenylation sites. Comparison of RACE and genomic sequences revealed that 15 genes contained introns in their 5' or 3' UTR regions. Seven genes had one intron and one had two introns in their 5' UTRs. Six genes had one intron and one had three introns in their 3'UTRs. The presence of introns in either 5' or 3' UTRs has also been indicated in the TAIR database [89]. Six out of eight genes which have introns in their 5' UTR according to our RACE results have also been annotated as having introns in the TAIR database, and similarly five out of seven genes which have introns in 3' UTR have also been so annotated in the TAIR database.

Sequence analysis of the RACE-PCR products revealed that the annotations of 12 NBS-LRR-encoding and related genes in the TAIR database were incorrect or at least inconsistent with our experimental data (Additional file 1). These genes had been annotated with incorrect transcript initiation or termination sites, different splicing sites, or extra exons. The RACE-PCR products validated the previous manual re-annotations for six genes [7]. The errors in the other six genes had not previously been detected. These six genes had different splicing events from those predicted. RACE-PCR data also revealed the presence of alternative splicing in the 5'UTR of At1g10920 and 3'UTR of At1g72860, in addition to the four genes which were indicated by MPSS data as exhibiting alternative splicing and had been confirmed by RACE-PCR and sequencing.

## Discussion

Our comprehensive expression analysis revealed that the majority of NBS-LRR-encoding and related genes in Arabidopsis were expressed at low levels and unexpectedly that many exhibited tissue-specific expression patterns (Additional file 1). The expression of some but not the majority of NBS-LRR-encoding and related genes was affected by treatments with defense signaling chemicals. The expression pattern of most of the previously uncharacterized NBS-LRR-encoding and related genes resembles that of known *R *genes and therefore is consistent with these genes also functioning in disease resistance.

### Consistency between different analytical approaches

The different analytical approaches varied in their sensitivity and accuracy, but the expression data obtained from each approach were generally consistent. Both MPSS and microarrays were the most efficient genome-wide transcript profiling methods and they correlated well. MPSS has several advantages over microarray analysis. MPSS is more sensitive and accurate, since MPSS provides quantitative assessment of the abundance of each transcript as opposed to the hybridization intensity generated for each probe set in the microarray analysis. MPSS is particularly advantageous for genes that are expressed at low levels and therefore tend to be more affected by background noise in microarray analysis. For example, both At5g45230 and At1g17600 were expressed at undetectable levels in microarray analysis of leaf tissue; however, based on MPSS data, At1g17600 was expressed clearly higher than At5g45230 in the leaf library (14 vs 0 TPM) (Additional file 1). MPSS can also distinguish multi-gene family members better than microarrays and therefore decrease cross-hybridization problems, which is more common in microarray analysis. Inconsistencies between MPSS and microarray data could have been due to cross-hybridization problems in the microarray analysis or due to some MPSS tags representing several gene family members that had high sequence similarity. In addition, sequencing errors in the current Arabidopsis genome assembly may have caused incorrect assignment of some MPSS tags and thus inaccurate determination of expression. While MPSS has the above advantages, its technical accessibility and high cost limit its widespread use, although these issues may be ameliorated with the latest sequencing technologies. Microarrays therefore remain a useful complement to investigate situations for which MPSS data do not exist until other high throughput sequencing technologies become available that allow affordable, in depth analysis of EST representation.

### Expression levels of NBS-LRR-encoding genes

The majority of NBS-LRR-encoding and related genes examined in this study were expressed at low levels in unchallenged plants similar to what has been observed for most cloned plant *R *genes. Significant changes in expression of most NBS-LRR-encoding and related genes including the known *R *genes were not detected during plant defense responses or treatments with two defense signaling molecules, SA or JA. Other recent RNA profiling experiments also failed to detect differential expression of *R *genes [90,91]. Similarly, in another microarray experiment performed to study gene expression changes during the resistance response, none of the Arabidopsis NBS-LRR-encoding genes showed significant expression changes during the plant defense response mediated by *RPS2*, *RPM1*, *RPS5*, or *RPS4 *(A. Bent et al., unpublished). This lack of induction of gene expression during plant defense response resembles that of most known plant *R *genes. Although it is still not clear how plant R proteins function in the plant defense response, it is clear that they act at an early step in defense signaling pathways, either as primary recognition molecules or accessory proteins [14-16,92-94]. Low levels of constitutive expression of R proteins are consistent with a constitutive ability to recognize the pathogen infection and induce downstream defense responses.

There are, however, several indications of transcriptional control of *R *gene expression. At least a subset of *R *genes are induced above their low levels of constitutive expression during the elicitation of basal resistance; the expression of *RPS2*, *RPM1*, and eight other NBS-LRR-encoding and related genes was induced by the bacterial flagellin peptide, flg22 [78]. This can be thought of increasing the general sensitivity of the plant to detect potential pathogens. Our analysis also revealed that fifteen NBS-LRR-encoding and related genes in wild-type Col-0, including the Col-0 homolog of *RPP4*, were induced 4 hours after treatment with SA; also several other genes were induced by SA in other accessions. Interestingly, out of the fifteen NBS-LRR-encoding and related genes induced by SA in Col-0, two genes also exhibited elevated expression induced by flg22. This overlap suggests interactions between plant basal defense response and SA signaling pathways. The MPSS data indicated that expression of many NBS-LRR-encoding and related genes was also affected by plant developmental stage or treatments with sucrose or the plant hormone ABA. In addition, our gene trap studies of a limited number of *R *gene related sequences demonstrated the induction of the expression of one TIR-NBS-encoding gene, At1g72910, by the SA homolog, BTH. A previous transcript profiling experiment in Arabidopsis also revealed the expression of several NBS-LRR-encoding and related genes was altered during defense response to cucumber mosaic virus strain Y; the expression of one TIR-NBS-LRR-encoding gene, At1g56510, and one TIR-X-encoding gene, At1g65400, was down-regulated, and that of two other TIR-X-encoding genes, At1g72940 and At1g72920, was induced [95]. These results all provide evidence for regulation of *R *gene expression during plant defense response and the induction of enhanced levels of defense-related surveillance in response to biotic challenge.

### Tissue specificity

An unexpected result from the current study was the frequent tissue-specific expression patterns exhibited by NBS-LRR-encoding and related genes. Both the MPSS data and the microarray data demonstrated that many NBS-LRR-encoding and related genes showed tissue specificity. Some genes were mainly expressed in aerial parts of plants, while some genes were specifically expressed in roots. Others appeared to be developmentally regulated. These patterns of differential expression suggest either that NBS-LRR-encoding and related genes function in resistance to a variety of pathogens that attack different parts of the plant, or that some NBS-LRR-encoding and related genes function in different plant biological processes. Previous to our studies there was little data on the tissue specificity of known *R *genes. All of the 14 known NBS-LRR-encoding *R *genes or their Col-0 homologs analyzed in our study exhibited tissue specific gene expression. Interestingly, the tomato NBS-LRR-encoding *R *gene, *I-2*, is expressed at the site of lateral root formation indicating that it might have a role in lateral root initiation in addition to disease resistance [38].

### Alternative transcripts

Alternative splicing was detected for several NBS-LRR-encoding genes. Twelve NBS-LRR-encoding genes showed evidence of alternative splicing based on the locations of MPSS tags and four of these genes, including the Col-0 homologs of two known *R *genes, *RPP5 *and *RPP4*, were confirmed by RACE-PCR and subsequent sequencing. The alternative transcripts of two genes (At1g63750 and At4g16860) encode truncated proteins containing only the majority of TIR domain and lacking both the NBS and LRR domains. The alternative transcript of At5g46270 encodes a truncated protein lacking most of the LRR domain, while alternative transcripts of At4g16950 encode TIR-NBS-LRR proteins with only the last few amino acids altered. The alternative splicing in At1g10920 and At1g72860 which was revealed by RACE-PCR occurs in the 5' or 3'UTR and therefore does not change the amino acid sequence; however, such alternative splicing could affect transcript stability and therefore the expression level. Alternative splicing has been reported for seven known TIR-NBS-LRR-encoding *R *genes and one CC-NBS-LRR-encoding *R *gene [46,49-55]. Based on alignments of genomic sequences with full-length cDNA and EST sequences, 1186 Arabidopsis genes in the TIGR database [96] have been annotated as undergoing splicing variation and have been classified into five different types of splicing variants. Although ten NBS-LRR-encoding or related genes are included in these 1186 genes, only one gene was identified as exhibiting alternative splicing by MPSS or RACE analysis in our studies. A recent extensive computational analysis [97] identified alternative splicing events in 4707 Arabidopsis genes including 16 NBS-LRR-encoding and related genes. Seven out of these 16 genes were also identified in TIGR database; however, only two out of these 16 genes were also detected as showing alternative splicing by MPSS or RACE analysis in our studies. This inconsistency may be due to RACE and MPSS tending to analyze sequences at the ends of transcript or due to the lack of sampling the appropriate tissues or conditions. Together, these data indicate that at least 22 TIR-NBS-LRR-encoding, one TIR-NBS-encoding, one divergent NBS-LRR-encoding, and eight CC-NBS-LRR-encoding genes exhibit alternative splicing in Arabidopsis.

The role of alternative splicing in plant *R *genes is unclear. The splice variants might interact with the full length R protein and have a regulatory role in disease resistance as has been suggested for the tobacco *N *and Arabidopsis *RPS4 *genes. This is similar to the role of alternative splicing in animal toll-like receptor (TLRs) [98]. The alternative transcript of the tobacco *N *gene is induced by challenge with tobacco mosaic virus and the ratio of the two transcripts appears to be critical for resistance [50,99]. The presence of both full-length and alternative transcripts of Arabidopsis *RPS4 *is necessary for the *RPS4*-mediated defense response [52,100]. However, the alternative transcripts of flax *L6 *and tomato *Bs4 *seem not to be important for the resistance that these genes mediate [49,54].

One of the TIR-NBS-encoding genes, At1g72910, may function in plant defense response as indicated by the induction of its expression by a SA analog, BTH. Similarly, six TIR-NBS-encoding genes, At1g17610, At1g66090, At1g72890, At1g72900, At1g72950, and At3g04210, may also function in plant defense response since their expression was affected by SA treatment in at least one of the seven Arabidopsis ecotypes studied. Two TIR-NBS-encoding genes, At1g72900 and At1g72940, may function in plant basal defense response since their expression was induced by flagellin. In the Arabidopsis Col-0 genome, there are 21 TIR-NBS-encoding genes that have similar structures as the alternative transcripts of the tobacco *N*, flax *L6 *or Arabidopsis *RPS4 *genes [9]. These might function similarly to the alternatively transcribed variants of TIR-NBS-LRR-encoding genes.

### Post-transcriptional regulation

*R *genes do not need to be induced at the transcriptional level in order to alter resistance against pathogens. There are likely to be multiple levels of negative regulation to prevent the inappropriate activation of R proteins in the absence of pathogen that would be deleterious to plants due to the high cost of the defense response and pathogen-independent cell death. There may also be feedback loops controlling the *R *gene expression and the extent of HR.

Our study analyzed steady state mRNA levels, which may not reflect protein abundance. Further work is required to identify whether there are differences in the polysomal fraction and what post-translational modifications occur. The expression of several *R *genes has been reported to be regulated at the post-transcriptional level. The transcript of Arabidopsis *RPM1 *remains at a low level before and after pathogen attack; however, the RPM1 protein is degraded during HR [101]. The expression of *Xa21 *gene transcript is independent of plant developmental stage, though the *Xa21*-conferred resistance is developmentally regulated [102].

Data from animal systems have demonstrated the involvement of the 5'UTR in controlling translation and tissue specific expression [103,104]. Several plant *R *genes contain introns and/or upstream open reading frames (uORFs) in their 5'UTR including barley *Mla6 *and Arabidopsis *RPP1-WsB *and *RPP1-WsC *[19,105]. Our RACE analysis revealed that fifteen out of the 81 NBS-LRR-encoding and related genes analyzed contained introns in their 5' or 3' UTRs. These 5'UTR features may be indicative of post-transcriptional regulation of these genes.

## Conclusion

Transcripts of most NBS-LRR-encoding and related genes analyzed were present at low levels in unchallenged plants. Many showed tissue specific expression patterns. Transcript levels of the majority of NBS-LRR-encoding and related genes were not altered during the plant defense response or by treatments with plant defense signaling molecules; however, the expression of several genes was altered and may be indicative of altered levels of surveillance by the plant. Our data are consistent with the primary function of the majority of NBS-LRR-encoding and related genes being plant resistance; however, this does not preclude their involvement in functions other than pathogen recognition.

Future studies on the significance of tissue specificity, the roles of alternative transcripts and the relationship between transcript and protein levels will likely be informative as will the characterization of the spectrum of genes induced downstream of each major clade of NBS-LRR-encoding genes.

## Methods

### Expressed Sequence Tag (EST) analysis

EST representation for each Arabidopsis NBS-LRR-encoding or related gene was obtained by searching the NCBI EST database using the predicted cDNA sequence or genomic sequence (plus 500 bp upstream and downstream of the predicted start and stop codons) of each NBS-LRR-encoding or related gene [7] using BLAST [106]. As of July 14^th^, 2006, this database contained a total of 622,792 Arabidopsis EST sequences including: short, single read cDNA sequences, cDNA sequences from differential display experiments and RACE analyses, and cDNA sequences from full-length cDNA clones from RIKEN (The Institute of Physical and Chemical Research, Japan) [107]. All Arabidopsis ESTs with matches of greater than 80% identity to NBS-LRR-encoding and related genes were investigated. EST representation was determined based on the alignment between ESTs and the genomic or cDNA sequence of the corresponding gene and usually showed > 97% sequence similarity. Each potential representative EST was compared against the complete Arabidopsis genomic and spliced sequences using TAIR BLAST tool [108] to confirm that it was the best match to the represented gene. The ESTs showing best match to a specific NBS-LRR-encoding or related gene but with sequence identity less than 97% due to obvious sequencing difficulties were also counted. The ESTs that showed the same level of sequence similarity to several closely related family members were counted for each represented gene. A similar analysis was also performed earlier in April 2002 except that FASTA [109] was used to search for sequence similarity. The NCBI EST database contained about 181,406 Arabidopsis sequences at that time.

### Massively Parallel Signature Sequencing (MPSS) data

MPSS provides a comprehensive assessment of gene expression by generating short sequence tags, each 17 to 20 bp long, produced from a defined position (usually the first *Sau*3A restriction site 5' to the polyadenylation site of a transcript) within each transcript [67,68]. The expression level of each gene in a sample is determined by counting the number of diagnostic sequence tags representing the transcript of a particular gene.

The DuPont MPSS database contained expression profiles generated from 22 Arabidopsis MPSS libraries. Half were constructed from Arabidopsis ecotype Columbia (Col-0) or Landsberg *erecta *(L*er*) tissues collected at different developmental stages and half were constructed from wild type or mutant Col-0 or L*er *ecotype plants treated with various chemicals or hormones including abscisic acid (ABA), dexamethasone (DEX), or sucrose (Table 3). Each library contained approximately one million 17 bp tags. Seventeen additional MPSS libraries were made from various Arabidopsis Col-0 tissues (callus, flower, leaf, silique, and root from wild-type or flowering mutants) or salicylic acid (SA) treated leaf tissues and displayed in the public MPSS database (Table 4, [74-76]). Each of these public libraries contained about two and half million 17 bp tags.

For the analysis of the DuPont MPSS data, searches were performed using the genomic sequence for each NBS-LRR-encoding or related gene plus 500 bp upstream and downstream of the predicted start and stop codons. In a few cases the additional flanking sequence overlapped adjacent genes with small intergenic regions; however, these were manually checked and did not contain expressed tags that could have biased the analysis. Each expressed tag was also compared against the complete Arabidopsis genomic and spliced sequences using TAIR BLAST tool [108] to confirm the correct match to the designated gene; only matches to the sense strand were used in the calculations of transcript abundance. The frequency of each tag was counted and then normalized in parts per million (PPM) to calculate the abundance of each transcript in the sample. The PPM values were adjusted (adjPPM) to account for potential sequencing errors (described in [9]). The possible alternative polyadenylations caused by alternative splicing and variable stop codons were predicted based on the tag location. For the Arabidopsis MPSS data generated by Meyers et al. [75,76], a basic query was performed for each gene based on gene "At" number (gene identifier) against the 17 MPSS libraries. To allow comparisons among libraries, the signature frequencies were normalized to transcripts per million (TPM). For genes associated with multiple expressed signatures, the sum of abundance for all expressed signatures, including the signatures with more than one hit to the Arabidopsis genome, was used to indicate the abundance of each transcript in the sample.

In order to investigate potential correlations between gene expression and phylogenetic position, the expression level and tissue specificity of each NBS-LRR-encoding or related gene was compared with the phylogenetic tree described previously [7]. To investigate potential correlations between gene expression pattern and chromosomal location, these NBS-LRR-encoding and related genes were sorted by their gene identifiers, which usually reflects their chromosomal locations, and then the expression pattern of NBS-LRR-encoding and related genes was visually compared to their locations on this sorted gene list.

### Microarray analysis

Concurrent global expression experiments using ATH1 Affymetrix arrays provided the opportunity to assess the expression patterns of NBS-LRR-encoding and related genes.

The experiment analyzing the response to applications of salicylic acid (SA) was described in [79]. Six-week old plants from seven *Arabidopsis thaliana *accessions (Col-0, Cvi-1, Est, Kin-0, Mt-0, Tsu-1, and Van-0) were sprayed with 0.3 mM SA in 0.02% Silwet L77. Plants treated with 0.02% Silwet L77 were used as controls. The aerial parts of the plants were harvested 4, 28 or 52 hours later. Each treatment and time-point was replicated three times. Gene expression levels were assayed using the Affymetrix ATH1 GeneChips. The ATH1 array contains approximately 22,000 genes including 152 probe sets representing 162 NBS-LRR-encoding and related genes based on Affymetrix annotation [110]. Raw data (CEL files) were imported into GeneChip^® ^Operating Software data base (GCOS). Transcript abundances for all probe sets on the Affymetrix ATH1 GeneChips array were analyzed using GCOS. GCOS was also used to assess the presence or absence of a given transcript (P, present; A, absent; M, marginal) for each probe set. To allow direct comparison between chips, raw signals were globally scaled so that the mean expression level of each array was equal to an arbitrary target intensity of 500. The scaled signals were then imported into Excel for further analysis.

Raw array data (CEL files) of another experiment [78] studying flagellin treated Arabidopsis plants were obtained from ArrayExpress [111]. In this experiment, the L*er *accession of Arabidopsis seedlings were treated with 10 μM flg22 peptide and plantlets analyzed 30 minutes after treatment. GCOS was used to extract the expression signal and assign a present or absent call for each gene represented by a probe set on the ATH1 array. The data was processed in the same manner as for the SA induction experiment: raw signals were globally scaled to a target intensity of 500 for direct comparison.

Microarray analysis was also performed on the same five total RNA samples used for generating five of the 17 public MPSS libraries. Gene expression levels were also assayed using the ATH1 arrays. Complementary RNA labeling, hybridization, and signal acquisition were performed according to the manufacturer's guidelines (Affymetrix, Santa Clara, CA). Affymetrix Microarray Suite version 5.0 (MAS 5.0) was used to control washing, scanning and data-preprocessing steps. Raw CEL files were then imported into GCOS and GCOS was used to extract the expression signals and assign a present or absent call. To allow direct comparison across chips, raw signals were globally scaled to a target intensity of 500.

All data analysis was subsequently performed using GeneSpring GX 7.3.1 software (Agilent Technologies, Santa Clara, CA). The raw output signals from GCOS, without scaling and normalization, were used as input for analysis in GeneSpring GX. The raw signals were first normalized using the 50th percentile of all measurements on a given chip and then the median measurement of each gene was adjusted to 1. In order to identify differentially expressed genes in response to SA or flagellin, a list of probe sets showing reliable and detectable expression was first established using several criteria. Probe sets were retained if they were called as present or marginal by GCOS in both replicates (or two out of three replicates) of at least one of the two comparison conditions, their coefficient of variation (CV) were less than 0.3 in at least one of the two comparison conditions, and they exhibited at least two fold changes. Starting from this list of genes, the genes exhibiting a significant expression change in a given treatment were identified as those genes passing the parametric test (Welch t-test) in GeneSpring GX, without assuming equal variances and with multiple testing correction (Benjamini and Hochberg False Discovery Rate), and a *P*-value threshold of 0.1. The log-transformed normalized signals were used when performing these parametric tests.

The expression level polymorphism between seven Arabidopsis accessions for each NBS-LRR-encoding gene was determined using ELP Finder tool [112]. Affymetrix ATH1 GeneChip probeset IDs were entered as query and the average expression value for each gene in a given accession with the standard error was returned. The results of pairwise t-tests between the accessions selected were also returned.

The possible correlation between MPSS and microarray data was analyzed using Spearman rank correlation test conducted in SAS9.1 (SAS Institute, Cary, NC). The MPSS and microarray data analyzed using this test was generated from the same leaf sample and only the 88 NBS-LRR-encoding and related genes that had unique representative Affymetrix probe sets and MPSS sequence tags were included.

### Plant materials and growth conditions

Arabidopsis Col-0 plants used for Rapid Amplification of cDNA ends (RACE) analysis were grown in soil (Premier Pro-Mix B mix) in controlled-environment chambers at 21°C with 50% humidity, on a 16 hr light/8 hr dark cycle, and under 100 to 120 μEi illumination.

The gene trap and enhancer trap lines (Landsberg *erecta *(L*er*) background) were obtained from Cold Spring Harbor Laboratory (CSHL) or Singapore Institute of Molecular Agrobiology (IMA) [85,86]. The seedlings from each gene trap line were first selected on 1/2 MS medium agar plates with 50 μg/ml kanamycin and then the surviving seedlings were transferred to soil. These plants were grown in controlled-environment chambers with the same conditions as above.

### Analysis of gene trap lines

The gene traps lines carried the β-glucuronidase (*uidA*) reporter gene at sequence-characterized positions [81-84]. The genomic sequences of 207 NBS-LRR-encoding and related genes were searched against the flanking sequence databases of Gene Trap lines generated at Cold Spring Harbor Laboratory (CSHL) [85] and *Ds *insertion lines generated at Singapore Institute of Molecular Agrobiology (IMA) [86] using BLAST [106]. For each trap line, tissue PCR [113] was performed to confirm the insertion site and determine the orientation of the inserted element by using a gene-specific primer and a *uidA *specific primer.

To study the induction of NBS-LRR-encoding gene expression, jasmonic acid (JA; 500 μM) was dissolved in 10% v/v ethanol and sprayed onto five-week old plants in sterile containers. Benzothiadiazole (BTH; 1.2 mM) was dissolved in water with a wettable powder carrier (same amount of carrier as BTH) and similarly sprayed onto the five-week old plants in sterile containers. Treatments with 10% v/v ethanol or the wettable powder carrier were used as controls for each experiment. Whole plants were collected at different time points from 0, 24, and 48 hrs, to 5 days after each treatment and then subjected to GUS histochemical staining and GUS activity assays. Each gene trap plant was also challenged with *P. syringae *pv. *tomato *strain DC3000 carrying *avrRpt2 *or *avrRpm1 *which caused a hypersensitive response (HR) in wild-type Col-0 and L*er *plants. The HR response was examined at 16 hours, 1 day and 2 days after pathogen infiltration and GUS staining was performed at the same time.

Histochemical staining for GUS activity was performed according to a slightly modified protocol from [114,115]. Plant tissues were immersed in the GUS staining solution (100 mM sodium phosphate (pH7.0), 2 mM K_3_Fe(CN)_6_, 2 mM K_4_Fe(CN)_6_, 10 mM EDTA, 0.1% (v/v) Triton X-100, 100 μg/ml chloramphenicol, with 1 mg/ml 5-bromo-4-chloro-3-indolyl-β-D-glucuronide cyclohexylammonium salt (X-gluc, Gold Biotechnology Inc., St. Louis, MO, U.S.A.). The plant tissues were incubated with GUS staining solutions at 37°C for 24 hrs.

GUS activity was quantified fluorometrically using 4-methylumbelliferone glucuronide (MUG, Sigma) as a substrate as described by Jefferson et al[114]. Reactions were performed in 200 μl of extraction buffer (50 mM NaPO_4 _pH7, 10 mM EDTA, 0.1% Triton X-100, 0.1% Sodium Lauryl Sarcosine, 10 mM β-mercaptoethanol) containing 1.1 mM MUG and stopped after 1 h incubation at 37°C by addition of 800 μl 0.2 M Na_2_CO_3_. The fluorescence was calibrated using 4-methylumbelliferone (MU, Sigma, St. Louis, MO, USA). Total protein was determined using Bradford reagents and BSA as a standard [116].

### Rapid Amplification of cDNA Ends (RACE)-PCR

RACE-PCR was performed with the Marathon™ cDNA Amplification Kit (Clontech, Mountain View, CA, USA) according to the manufacturer's instructions. Total RNA was extracted from the leaves of about four-week old wild-type Arabidopsis Col-0 plants, seven-day old seedlings, or flower tissues using the TRIzol procedure (Invitrogen, Carlsbad, CA). Template mRNA was then purified from total RNA using Dynabeads^® ^Oligo (dT)_25 _(Dynal Biotech, Lake Success, NY, USA). RACE-PCR products were cloned into the pCR^®^2.1-TOPO^® ^vector (Invitrogen, Carlsbad, CA) and sequenced. Sequence data were analyzed using BLAST and Sequencer 3.1 (GeneCodes, Ann Arbor, MI). All RACE sequences were deposited into NCBI GenBank under accession numbers from ES444179 to ES444640 and from EX654484 to EX654486.

### Reverse Transcription-PCR (RT-PCR)

RT-PCR was carried out using Advantage™ RT-for-PCR Kit (Clontech, Mountain View, CA) according to the manufacturer's instructions. Primers were designed to amplify regions containing an intron in order to distinguish genomic DNA contamination from gene transcripts.

## Authors' contributions

XT conducted the majority of the experiments, analyzed the data and drafted the paper. BCM assisted in the experimental design, data analysis and writing. AK provided bioinformatics support for analysis of EST and genomic Arabidopsis sequences and data visualization. MALW and DAStC contributed the microarray experiments on the Arabidopsis accessions and the SA treatment as well as assisted in data interpretation. MM contributed MPSS data. AFB contributed to the experimental design and writing. RWM contributed to the overall experimental design, data interpretation, and writing of the paper.

## Supplementary Material

Additional file 1 Detailed summary of expression analyses of NBS-LRR-encoding and related genes. Worksheet 1A provides the data structure of worksheet 1B and 1C. Worksheet 1B provides full expression data of CC-NBS-LRR-encoding and related genes. Worksheet 1C provides full expression data of TIR-NBS-LRR-encoding and related genes. Data is also available online [73]. Click here for file

Additional file 2 The 38 NBS-LRR-encoding and related genes showing altered expression in at least one of three time points (4, 28, or 52 hrs) post treatment with salicylic acid in multiple accessions of Arabidopsis or following treatment with flagellin in wild-type L*er *(raw data from [79] and [78], respectively). Fold change values are presented for each treatment x control comparison. Red indicates statistically significant up-regulation; blue indicates statistically significant down-regulation. Columns 5 – 13 display data from different ecotypes treated with SA. Column 14 contains data from L*er *treated with flagellin. NBS-LRR-encoding and related genes that did not show statistically significant changes are not shown. Click here for file
